# Dual burden of malnutrition among mother-child dyads in Mozambique: Findings from the Demographic and Health Survey 2022–2023

**DOI:** 10.1371/journal.pone.0361129

**Published:** 2026-10-21

**Authors:** Nahin Shakurun, Fernanda André, Nazeem Muhajarine

**Affiliations:** 1 Department of Community Health and Epidemiology, College of Medicine, University of Saskatchewan, Saskatoon, SK, Canada; 2 Saskatchewan Population Health and Evaluation Research Unit, Saskatoon, SK, Canada; Universidade de Sao Paulo Faculdade de Saude Publica, BRAZIL

## Abstract

**Introduction:**

Nutritional challenges are a global public health concern, especially among children under five in sub-Saharan African countries. The coexistence of an overweight/obese mother and an underweight child in the same household is recognized as a dual burden of malnutrition (DBM). This study estimated the prevalence, identified associated factors, and assessed socioeconomic inequalities in DBM among mother-child dyads in Mozambique.

**Methods:**

We used nationally representative, cross-sectional data from the Mozambique Demographic and Health Survey 2022–2023 (n = 3,605 mother-child dyads). Child undernutrition and maternal BMI status were calculated using the World Health Organisation (WHO) standard guidelines. The outcome variable, DBM, was defined as the presence of child undernutrition condition (stunting, wasting or underweight) with corresponding maternal overweight/obesity. Multivariable binary logistic regression, Erreygers’s concentration index, and concentration curve analyses were performed, accounting for the complex survey design, to determine associated factors and social inequalities.

**Results:**

The prevalence of DBM was 5.5% [95% CI: 4.6, 6.6]. Mothers aged ≥ 34 years [aOR (95% CI): 4.01(1.44, 11.14); p < 0.05] and mothers with four or more children [aOR (95% CI): 2.68(1.29, 5.57); p < 0.05] had higher odds of DBM. Rural residence and using unimproved toilet facilities (latter an indicator) were also significantly associated with DBM. Additionally, maternal age modified the association between women’s empowerment and mother-child DBM. Women aged 15–19 years at the lowest and highest empowerment levels were more likely to experience DBM than women 20 years or older. A positive and statistically significant concentration index indicates that wealth-related inequalities exist, with DBM more concentrated among wealthier mother-child dyads.

**Conclusion:**

Our study highlights the occurrence of household-level DBM in Mozambique. These findings emphasize the need for targeted interventions addressing social and economic inequalities, including poor sanitation, a marker of broader household deprivation. Prioritizing integrated maternal-child nutrition interventions within national strategies is essential to improving equity in nutrition and health outcomes.

## Introduction

Until recently, undernutrition and overnutrition were traditionally considered two distinct public health concerns. However, global evidence now demonstrates that both undernutrition and overnutrition can coexist within the same community, household, or individuals, a phenomenon commonly referred to as the dual burden of malnutrition (DBM) [1–4]. At the household level, DBM refers to the simultaneous presence of undernutrition in one household member and overweight or obesity in another [5,6]. This coexistence is increasingly observed in low-and middle-income countries (LMICs), where nutrition transitions, urbanisation, globalisation, and changing lifestyles have occurred alongside persistent food insecurity and infectious diseases [7–9].

DBM is a significant public health issue and is closely linked to Sustainable Development Goals (SDGs), particularly target 2, which aims to eradicate hunger and ensure that all people, particularly those living in poverty and people in other vulnerable conditions, including infants and children under five, have access to sufficient and nutritious food [10]. Despite substantial progress in reducing undernutrition globally, malnutrition continues to affect millions of women and children. In 2020, undernutrition contributed to approximately 45% of deaths amongst children under five, while estimated 149 million children were affected by stunting and more than 49 million by wasting [11,12]. At the same time, overweight and obesity are increasing in LMICs.

Women of reproductive age (WRA) are particularly affected by this changing nutritional landscape. In Africa, underweight among WRA has declined considerably over the last few decades, but remains prevalent, while overweight and obesity continue to increase across LMICs [13]. Studies have reported that 27% of women in Mali and 63% in Ethiopia are overweight or obese [14]. Similarly, the magnitude of overweight and obesity among women was 28.1% and 7.7% in Kenya, respectively, while corresponding estimates in Ghana were 21% and 12% [15,16]. Maternal undernutrition has also been reported in Ghana, Tanzania, Kenya, and Ethiopia, with prevalence estimates ranging from 4% to 25.5% [15–18]. Maternal nutritional status is important because WRA experiencing DBM may be at increased risk of adverse maternal outcomes for themselves and for their offspring during preconception, pregnancy, and breastfeeding [19–21]. These patterns demonstrate the complex nutritional challenges facing women and children in the region.

Mozambique, a country in the southeastern sub-Saharan Africa, continues to experience a high burden of malnutrition despite national and international efforts to improve food security and nutritional outcome. A meta-analysis of demographic and health surveys conducted to assess DBM in LMICs, revealed that Mozambique has one of the highest rates of undernutrition amongst children under five, estimated at 43% [22]. Although DBM has been documented in several LMICs, nationally representative evidence on mother-child dyads and socioeconomic inequalities in Mozambique remains limited. In particular, the coexistence of maternal overweight or obesity and child undernutrition within the same household has not been sufficiently examined using nationally representative data.

Several sociodemographic, socioeconomic, and environmental factors may influence the nutritional status of women and children [1,23]. Factors such as household wealth, place of residence, maternal age, education, employment, marital status, and access to healthcare may affect food security, healthcare utilisation, dietary practices, and other behaviours related to maternal and child nutrition [24,25]. Women’s empowerment may also play an important role because greater decision-making power, control over resources, mobility, and participation in household decisions may improve women’s access to nutritious foods, healthcare, and other resources that benefit both mothers and children [26]. However, the effects of empowerment may vary according to age, education, employment, household wealth, and other social conditions. Assessing women’s empowerment may therefore help identify pathways through which socioeconomic circumstances influence DBM and may indicate groups that require more targeted interventions.

In addition to identifying factors associated with DBM, understanding its socioeconomic distribution is important for developing equitable nutrition interventions. Regression analyses estimate associations between individual determinants and DBM but do not directly describe how DBM is distributed across the socioeconomic hierarchy. Concentration curves and the Erreygers’s concentration index are therefore appropriate to assess wealth-related inequalities in DBM, providing complementary information on its socioeconomic distribution alongside the factors identified through regression analyses.

Therefore, this study aimed to estimate the prevalence of DBM among mother-child dyads in Mozambique, identify associated factors, and assess socioeconomic inequalities in DBM using nationally representative data from the Mozambique Demographic and Health Survey 2022–2023. We also examined women’s empowerment as a potential determinant and effect modifier of DBM.

## Methods

### Data source

The Mozambique Demographic and Health Survey (DHS) 2022–23 dataset was used for this study [27]. It was a nationwide cross-sectional survey, encompassing ten provinces (Niassa, Cabo Delgado, Nampula, Zambezia, Tete, Manica, Sofala, Inhambane, Gaza, and Maputo), and the city of Maputo, which holds provincial status as the country’s capital city. Data collection employed a two-stage stratified sampling design. In the first stage, 619 enumeration areas (EAs) were selected with probability proportional to size based on the 2017 IV General Census of Population and Housing (IV RGPH). In the second stage, 26 households were systematically selected with equal probabilities from each EA, resulting in 16,045 selected households. All women aged 15–49 years who were usual residents or visitors in the household the night before the interviews were eligible for the interview.

For this analysis, we have used the children recode (MZKR) data file. Among women with at least one child under five years (n = 9,289), currently pregnant women were excluded from the study because maternal BMI cannot be appropriately assessed during pregnancy. Anthropometric measurements were available for a subsample of eligible mother-child pairs, resulting in 3,605 mother-child dyads included in the final analysis. Details of the participant selection process for this analysis are presented in supplementary file (S1 Fig in S1 File).

### Variables

#### Outcome variable.

The outcome variable was dual burden of malnutrition (DBM). DBM was defined as the coexistence of maternal overweight/obesity and child undernutrition, indicated by stunting, wasting, or underweight. Although these indicators represent distinct manifestations of child undernutrition with potentially different underlying determinants, they were combined to capture the overall occurrence of maternal overnutrition coexisting with any form of child undernutrition within the same household. In this study, participants were categorized as experiencing DBM if the child was experiencing stunting, wasting or was underweight and the mother was overweight/obese using WHO standards [28].

Anthropometric data on height and weight, collected in the survey, were used to measure the nutritional status of children and mothers [27]. For DHS data, the WHO 2006 children growth standard was used to calculate a standard deviation (SD) z-score [29]. Stunting, wasting, and underweight conditions for children were defined if height-for-age, weight-for-height, and weight-for-age z-scores below −2 SD of the WHO child growth standards median, respectively [29]. The measurements were considered invalid if height-for-age and weight-for-age z-scores below −6 SD and weight-for-height z-scores below −5 SD [29]. The prevalence of stunting, wasting, underweight was 31.9%, 3.52%, and 13.16%, respectively. Separate regression analyses for each child undernutrition indicator were not conducted, as these analyses were beyond the scope of the present study, which focused on identifying factors associated with the coexistence of maternal overnutrition and child undernutrition within the same mother–child dyad.

Maternal body mass index (BMI) was calculated as weight in kilograms (kg) divided by height in square meters. Maternal BMI was categorized into three groups: Underweight (BMI < 18.5 kg/m^2^), Normal (BMI 18.5–24.9 kg/m^2^), and Overweight (BMI > 24.9 kg/m^2^) [30]. A dichotomous variable was then created and labeled as “1” for DBM present (coexistence of maternal overweight and child stunting, wasting, or underweight) and “0” for DBM absent.

DBM can also occur in the reverse form (i.e., underweight mother and overweight/obese child), this form was not examined due to its low prevalence in this data [underweight mothers (5.02%, n = 181) and overweight/obese child (0.8%, n = 28)], in the study sample, which limits statistical power for meaningful analysis.

#### Independent variables.

The selection of independent variables was guided by the theoretical framework proposed by Hien and Hoa (2009) (S2 Fig in S1 File), relevant literature, and the presumed temporal and theoretical linkage between the explanatory variables and DBM [31–33]. The framework conceptualizes how DBM can be influenced by socio-demographic, maternal, environmental, and proximal factors. Independent variables considered in this study included maternal characteristics (age, education, marital status, employment status, number of children born alive), women’s empowerment index, and household characteristics.

To generate women’s empowerment index, we have used a modified version of recently developed Survey-based Women’s Empowerment (SWPER) Global index [34]. There were overlaps between the original SWPER index and our study variables, so we have decided to use a modified version of this scale. In this modified version we have used a set of 12 variables that capture all three distinct areas of women empowerment: decision making, social independence, and attitude towards violence. Instead of creating three separate women empowerment index scores, we have created a single composite empowerment score using principal component analysis and further categorized into five-quintiles similar to the household wealth index. Details of these variables and their categorization are presented in the supplementary S1 Table in S1 File.

Several household related variables such as place of residence (urban or rural), household wealth status, source of drinking water, toilet facilities and region were included in this analysis. The DHS household wealth index is a composite measure of living standards derived using principal component analysis of household assets, dwelling characteristics, and access to basic services; the score is grouped into five quintiles from poorest to richest [21]. Drinking- water sources were classified as improved or unimproved, and sanitation facilities were classified as improved or unimproved according to WHO definitions [20]. These variables were indicators that capture the overall household socioeconomic condition and environmental context in which maternal and child nutritional outcomes occur.

We also evaluated some child-related variables- child’s age, sex, birth weight, history of recent illness (two weeks prior to data collection), such as fever, cough, or diarrhoea. Details of these variables and their categorization are presented in the supplementary file (S2 Table in S1 File).

### Statistical analysis

We followed “Guide to DHS statistics-8” for this analysis [29]. The sample characteristics were presented with frequencies and percentages of the study variables. To identify the factors associated with DBM, bivariate logistic regression analyses were first conducted, and variables with a p-value <0.20 were considered for inclusion in the multivariable model. A multivariable binary logistic regression model was then constructed using forward selection approach. Starting with an empty model, candidate variables were added one at a time in order of statistical significance. After each addition, variables were retained in the model if they remained statistically significant at p < 0.05 or if their inclusion changed any existing coefficient by more than 20%, indicating potential confounding. The final model included maternal age, number of children, age of the child, women’s empowerment, place of residence, and type of toilet facilities. Rest of the variables did not meet the forward selection entry criteria and did not significantly or meaningfully confound the associations of the retained variables. Final results were reported as adjusted odds ratios (aOR) with 95% confidence intervals (CIs). Model fitness was assessed using the Hosmer–Lemeshow Goodness-of-Fit test. Multicollinearity among key explanatory variables was assessed using the variance inflation factor (VIF). All VIF values were below 5, indicating no evidence of problematic multicollinearity among the independent variables. Pre-specified effect-modification terms between women’s empowerment and maternal age, education, and employment status were tested using product term in the multivariable logistic regression model. Statistical significance was assessed based on the p-values of the corresponding interaction coefficients, with p < 0.05 indicating a statistically significant effect-modification. The significant effect-modification between empowerment and maternal age was retained and reported, whereas effect-modification between empowerment and maternal education and between empowerment and employment status were not significant and were therefore not retained in the final model.

The Concentration Index (CI) and Concentration Curve (CC) approaches were followed to identify household wealth and women empowerment-related inequalities in DBM [19]. The value of CI ranges between +1 to −1, with a zero-value indicating no socioeconomic inequalities. A negative value indicates unequal concentration of DBM among the poor (pro-rich inequalities), which can be visualized in CC as the segment below the 45-degree reference line and vice-versa (pro-rich inequality; CC above the 45-degree reference line) [35]. Because DBM is a binary outcome, Erreygers’s correction approach was applied to account for the bounded nature of the outcome and to obtain a normalized CI ranging between +1 to −1 [36]. This approach was also followed in previous research examining socioeconomic inequalities in binary health outcomes [37,38].

Data were analysed using Stata statistical software version 18.0 [39]. Sampling weights were applied to ensure representativeness of the sample. Statistical significance was considered at the p < 0.05 level.

### Ethics declaration

This study used data from Mozambique Demographic and Health Survey 2022–2023. The survey was approved by National Bioethics Committee for Health (CNBS) and ICF international institutional review board (IRB). Further approval for this study was not required since data is freely available in the public domain.

## Results

### Sample characteristics

Table 1 shows characteristics of the mother-child dyads. Majority of mothers (67.3%, n = 2398) were aged 20–34 years. Nearly half of the mothers (48.2%, n = 1696) had completed primary education, most of them were not employed (72.8%, n = 2435), married (82.7%, n = 2941), and multiparous (83.7%). Child characteristics showed a relatively even distribution across the age groups from 0–59 months, with a slightly higher proportion in the 13–24 months (21.4%, n = 772). Nearly equal distribution was observed among male (48.4%, n = 1787) and female (51.6%, n = 1818) children. Household characteristics indicated predominantly rural residence (71.2%, n = 2481). Regional distribution varied considerably, with the highest representation from Nampula (27.1%, n = 487) province.

**Table 1:** Distribution of sample characteristics: Mother-child dyads from Mozambique Demographic and Health Survey, 2022-2023.

Participants characteristics	Frequency	Weighted proportion (%)
**Maternal characteristics**		
**Maternal age**		
15-19 years	381	9.6
20-34 years	2398	67.3
34 and above	826	23.1
**Maternal educational status**		
No formal education	1026	31.2
Primary	1696	48.2
Secondary	817	19.4
Higher	66	1.2
**Employment status**		
Not working	2435	72.8
Working	1170	27.2
**Marital status**		
Never in union	213	5.1
Married/Living with partner	2941	82.7
Other (Widowed/Divorced/Separated)	451	12.2
**Number of children born alive**		
1	629	16.3
2-3	1380	37.7
4 or more	1596	46.0
**Women Empowerment Index (n = 2941)**		
Lowest (1^st^ quintile)	589	23.9
Lower (2^nd^ quintile)	610	23.9
Moderate (3^rd^ quintile)	834	27.9
High (4^th^ quintile)	598	16.8
Highest (5^th^ quintile)	310	7.5
**Child characteristics**		
**Age of the child**		
<6 months	515	14.4
6-12 months	414	11.0
13-24 months	772	21.4
25-36 months	675	19.2
37-48 months	651	17.4
49-59 months	578	16.6
**Sex of the child**		
Male	1787	48.4
Female	1818	51.6
**Birth weight**		
Low birth weight (<2.5 kg)	188	4.7
Normal (2.5–4.5 kg)	1316	33.1
High birth weight (>4.5 kg)	815	26.5
Not applicable	1286	35.7
**History of recent illness**		
**Fever**		
No	3150	89.1
Yes	455	10.9
**Cough**		
No	3133	90.5
Yes	472	9.5
**Diarrhoea**		
No	3215	91.0
Yes	390	9.0
**Household characteristics**		
**Place of residence**		
Urban	1124	28.8
Rural	2481	71.2
**Household wealth status**		
Poorest (1^st^ quintile)	784	26.8
Poorer (2^nd^ quintile)	670	22.2
Middle (3^rd^ quintile)	815	19.1
Richer (4^th^ quintile)	743	19.0
Richest (5^th^ quintile)	593	12.9
**Source of drinking water (n = 3564)**		
Improved	2241	56.3
Not improved	1323	43.7
**Type of toilet (n = 3564)**		
Improved	920	26.0
Not improved	2644	74.0
**Region/Provinces**		
Niassa	428	8.7
Cabo Delgado	479	6.8
Nampula	487	27.1
Zambezia	279	17.5
Tete	342	10.4
Manica	381	7.6
Sofala	361	7.0
Inhambane	229	3.4
Gaza	258	3.7
Maputo	203	5.7
Maputo city	158	2.1

### Prevalence of dual burden of malnutrition in mother-child dyads in Mozambique

Table 2 shows the pattern of malnutrition among mothers and children in Mozambique. Maternal malnutrition indicators showed that while most mothers (74.3%; 95% CI: 71.9, 76.5) had normal BMI, 6.2% (95% CI: 4.7, 8.2) were underweight and 19.5% were overweight/obese. Among children, more than one-third of the children (37.3%; 95% CI: 35.0, 39.8) had at least one form of undernutrition (stunting, wasting, or underweight). The combined mother-child nutritional profile demonstrated that 48.7% (95% CI: 46.2, 51.1) of the dyads had no malnutrition, while 31.8% (95% CI: 29.4, 34.3) of the dyads exhibited child undernutrition without maternal overweight (BMI: normal/underweight). Maternal overweight without child undernutrition was present in 14.0% (95% CI: 12.4, 15.8) of the dyads. Notably, though, 5.5% (95% CI: 4.6, 6.6) of the pairs demonstrated DBM, characterized by concurrent maternal overweight/obese condition and child undernutrition (either stunting, underweight, or wasting) in the same household. Among the provinces, Maputo city has the highest prevalence of DBM and Sofala had the lowest.

**Table 2:** Prevalence of nutritional burden among mothers and children in Mozambique: DHS 2022-2023.

Characteristics	Frequency	Weighted proportion (%) [95% CI]
**Maternal BMI status**		
Underweight	181	6.2 [4.7, 8.2]
Normal	2632	74.3 [71.9, 76.5]
Overweight/Obese	792	19.5 [17.6, 21.6]
**Undernutrition condition of children**		
No	2362	62.7 [60.2, 65.0]
Yes*	1243	37.3 [35.0, 39.8]
**Combined Nutritional status of mother-child dyads**		
Normal nutritional status	1762	48.7 [46.2, 51.1]
Maternal overweight, no child undernutrition	600	14.0 [12.4, 15.8]
Child undernutrition, no maternal overweight	1051	31.8 [29.4, 34.3]
DBM (Maternal overweight and child undernutrition present)	192	5.5 [4.6, 6.6]
**Dual burden of malnutrition**		
No	3413	94.5 [93.4, 95.4]
Yes	192	5.5 [4.6, 6.6]
**Regional distribution of DBM**		
Niassa	25	6.4 [4.2, 9.7]
Cabo Delgado	25	5.6 [3.0, 10.3]
Nampula	29	6.2 [4.2, 8.9]
Zambézia	19	5.5 [3.2, 9.3]
Tete	19	5.1 [3.0, 8.6]
Manica	24	6.8 [4.7, 9.6]
Sofala	6	1.9 [0.1, 4.3]
Inhambane	9	3.8 [2.0, 7.1]
Gaza	14	5.9 [3.3, 10.2]
Maputo	10	4.4 [2.0, 9.4]
Maputo city	12	7.7 [4.6, 12.8]

*either stunting/wasting/underweight.

### Factors associated with the dual burden of malnutrition among mother-child dyads in Mozambique

Results from bivariate analysis are presented in supplementary file (S3 Table in S1 File). Findings from the most parsimonious multivariable logistic regression model suggest that maternal age, number of children, age of the child, place of residence, and type of toilet facilities were significantly associated with DBM (Table 3).

**Table 3:** Results from final multivariable binary logistic regression model for factors associated with dual burden of malnutrition among mother-child dyads in Mozambique: Demographic and Health Survey, 2022-2023.

Characteristics	Dual burden of malnutrition	
	**aOR[95% CI]**	**p-value**
**Maternal characteristics**		
**Maternal age**		
20-34 years	Ref	
15-19 years	7.33[0.34, 156.16]	0.20
34 and above	4.01[1.44, 11.14]	**<0.05**
**Number of children born alive**		
1	Ref	
2-3	1.94[0.97, 3.87]	0.06
4 or more	2.68[1.29, 5.57]	**<0.05**
**Women Empowerment Index**		
Highest (5^th^ quintile)	Ref	
Lowest (1^st^ quintile)	1.70[0.69, 4.20]	0.25
Lower (2^nd^ quintile)	0.96[0.39, 2.41]	0.94
Moderate (3^rd^ quintile)	1.62[0.66, 3.97]	0.29
High (4^th^ quintile)	1.64[0.71, 3.78]	0.25
**Age of the child**		
<6 months	Ref	
6-12 months	0.57[0.20, 1.63]	0.29
13-24 months	1.76[0.88, 3.46]	0.12
25-36 months	1.95[1.00, 3.79]	**<0.05**
37-48 months	2.17[1.03, 4.61]	**<0.05**
49-59 months	1.61[0.76, 3.39]	0.21
**Place of residence**		
Urban	Ref	
Rural	0.51[0.33, 0.79]	**<0.05**
**Type of toilet (n = 3564)**		
Improved	Ref	
Not improved	0.60[0.38, 0.91]	**<0.05**

Note: Significant p-value <0.05 are marked in bold. The Hosmer-Lemeshow Goodness-of-Fit test significance at p = 0.93 indicated good fit of the model.

Mothers aged >34 years were 4 times more likely to have DBM [aOR (95% CI): 4.01(1.44, 11.14); p < 0.05] compared to those aged 20–34 years. The likelihood of DBM increased with the number of children born. Mothers with 4 or more children had significantly higher odds of experiencing DBM, with an odds ratio of 2.68 [aOR(95% CI): 2.68(1.29, 5.57); p < 0.05], compared to mothers with one child. Furthermore, children aged 25–36 months [aOR (95% CI): 1.95 (1.00, 3.79); p < 0.05] and children aged 37–48 months [aOR (95% CI): 2.17(1.03,4.61); p < 0.05] were more likely to be classified as DBM compared to children <6 months.

Compared with mother-child dyads residing in urban areas, those residing in rural areas had lower odds of DMB [aOR (95% CI): 0.51 (0.33, 0.79); p < 0.05]. Similarly, dyads living in households with unimproved toilet facilities had lower odds of DBM [aOR (95% CI): 0.60 (0.38–0.91); p < 0.05] compared to those living in households with improved toilet facilities.

A significant effect modification was observed between women’s empowerment and maternal age (p < 0.05), indicating that the probability of experiencing DBM associated with empowerment levels varied across different age groups and empowerment levels. Younger mothers, aged 15–19 years, with either lowest or highest empowerment levels were more likely to experience DBM compared to mothers aged 20–34 years with highest empowerment level (Fig 1).

**Fig 1:**
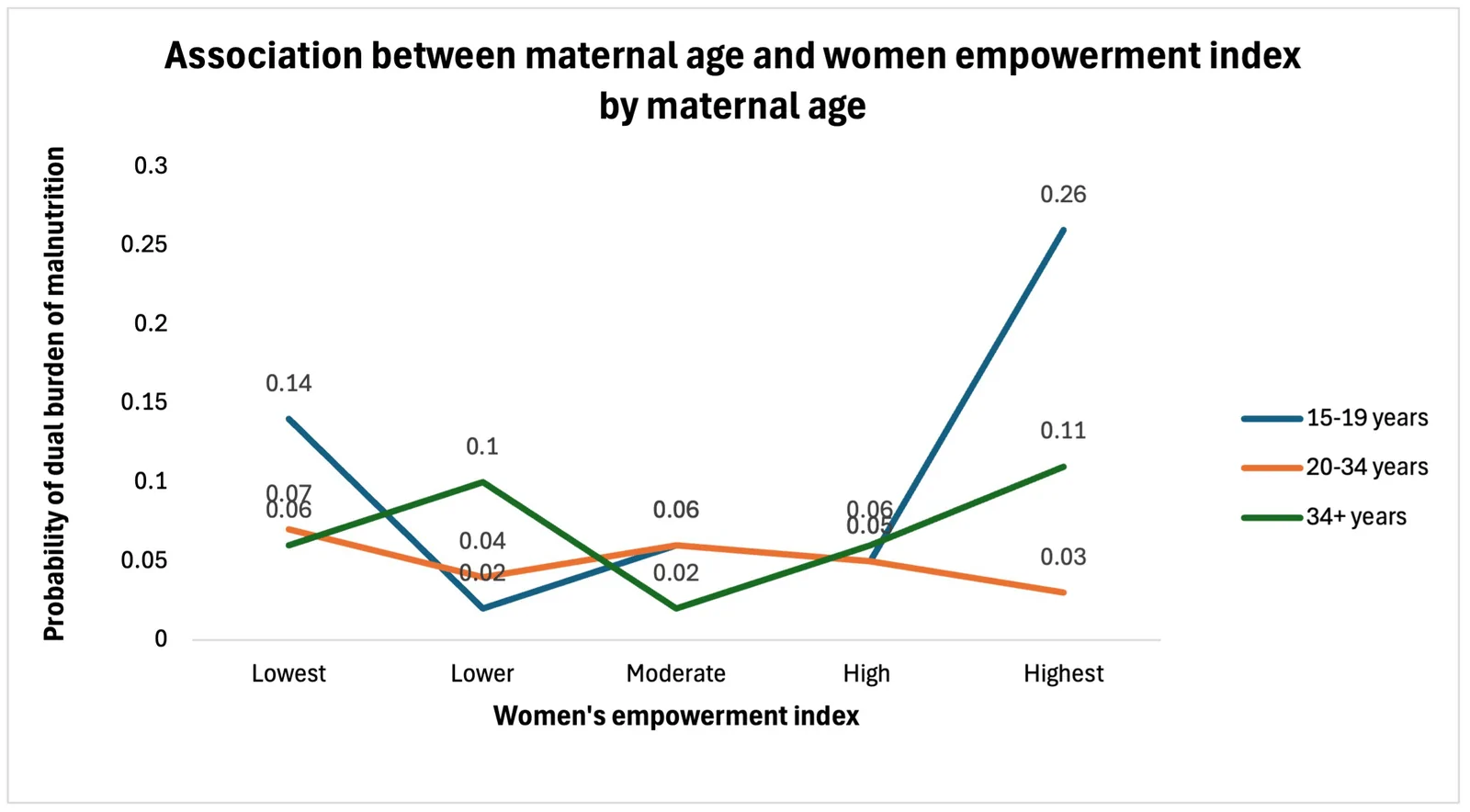
Effect modification plot showing association between women’s empowerment and DBM modified by maternal age groups.

### Inequalities in dual burden of malnutrition

The household wealth and women’s empowerment-related inequalities in DBM among mothers and children in Mozambique are presented in supplementary file (S4 Table in S1 File). A significant wealth-related disparity was observed based on Erreygers’s concentration index (ECI) (ECI (SE): 0.05(0.01); p < 0.001). The positive value of ECI indicates that the inequality in DBM was observed among higher wealth quintiles. The Concentration Curves (CC), showing that the estimated curve lies below the line of equity, indicating the presence of wealth related inequalities in DBM. These findings indicate that wealth-related inequality in the distribution of DBM was presented at the population level, but household wealth was not an independent predictor of DBM after adjustment for other covariates included in the regression model. In contrast, ECI for women’s empowerment index was not significant for DBM (S3 Fig in S1 File).

## Discussion

This study has systematically identified sociodemographic factors and social inequalities associated with dual burden of mother-child overweight and malnutrition in the same household, using nationally representative data from Mozambique. We found that maternal age, number of children born alive, age of the child, place of residence, and type of toilet facilities were associated with DBM.

In our study, the prevalence of DBM was approximately 5.5% among mother-child dyads. This finding is consistent with evidence from sub-Saharan Africa showing that maternal overweight and child undernutrition increasingly co-occur within households, reflecting the region’s ongoing nutrition transition [40–42]. For example, a national DHS‑based analyses in Burkina Faso has estimated prevalence near 4.9% [43]. Another study that analysed data from 26 DHS surveys across African countries examined multiple forms of DBM and reported prevalence estimates ranging from 5 to10% across different countries [44]. Moreover, a recent review found a substantial burden of double malnutrition among African children, with stunting prevalence of 37.9–57.7%, wasting of 10.5–15.5%, and underweight of 25.0–36.4%. These findings underscore the persistent implications of malnutrition for child health, growth, development, and future non-communicable disease risk [45].

In our study, 74.2% of mothers had a normal BMI, while 6.2% were underweight and 19.5% were overweight or obese. The proportion of mothers with a normal BMI in our study was higher than that reported in Sierra Leone (62.2%). Although BMI has recognized limitations as a measure of body composition, [46,47] it remains the standard anthropometric measure used in DHS surveys and allows comparison with previous population-based studies [47–50].

Consistent with prior work, our findings support the view that the drivers of DBM extend beyond the health sector and reflect broader social, economic, and cultural conditions. In many African settings, larger body size is associated with beauty, prosperity, and good health, which may contribute to higher acceptance and prevalence of overweight and obesity among women [51]. Poverty, hunger, and disease linked to insecure livelihoods, poor living conditions, limited education, and inadequate access to healthcare and nutritious food, remain central contributors to malnutrition in the region [40].

Maternal age was associated with household DBM, with older mothers having greater odds of DBM than mothers aged 20−34 years. These findings are consistent with the previous studies reporting a higher likelihood of experiencing DBM among mothers aged over 35 years (relative risk ratio (RRR): 4.18; 95% CI: 4.05–4.31) [52]. For example, Sunuwar et al. (2020), who reported that mothers above 35 years old were found to be at higher risk of experiencing DBM [53]. Furthermore, as age increases, the risk of diseases, especially dietary-related non-communicable diseases (NCDs) rises significantly, heightening the potential for nutrition-related health problems while child undernutrition co-exist in the same household.

In our study, over one-third of the children were affected by some form of undernutrition (stunting, underweight, or wasting). Children aged 25–36 months and 37–48 months had higher odds of DBM than children younger than 6 months. This finding is consistent with evidence from sub-Saharan Africa showing that children older than 6 months are more likely to experience DBM, with the highest risk of an overweight/obese mother-stunted child dyad observed among children aged 13–24 months and the highest risk of an overweight/obese mother-underweight child dyad among children aged 37–59 months [32]. A recent multicountry study in sub-Saharan Africa similarly reported a higher likelihood of DBM among children aged 2 years or older than among younger than 2 years (adjusted-OR: 2.12; 95%CI: 1.91–2.37) [54]. This pattern may reflect the period after complementary feeding begins, when inadequate dietary diversity, inappropriate feeding practices, and repeated infections can contribute to growth faltering, while maternal overweight persists within the household.

Further analysis also showed that the place of residence was associated with DBM. This finding is consistent with a multicountry study of 30 sub-Saharan African countries, which reported higher odds of household DBM in urban than rural areas (OR: 1.24; 95% CI: 1.06–1.46) [55]. The same study found higher odds of maternal overweight in urban areas, whereas child stunting was more common in rural settings, suggesting that urban food environments and sedentary lifestyles may increase maternal overweight while rural conditions continue to expose children to undernutrition [7,55]. Similarly, a national survey in Bangladesh reported overweight/obesity among 53% of urban women compared with 33% of rural women [56].This pattern may reflect greater physical mobility and burden among rural women engaged in farming, food preparation, and informal trading, which may reduce weight gain [25,57]. In contrast, urban women may have greater access to energy-dense, highly-processed packaged foods and more sedentary lifestyles, increasing their risk of overweight and obesity [52].

The association between adequacy of toilet facilities and DBM requires cautious interpretation. The lower odds of DBM observed among households with unimproved toilet facilities should not be interpreted as a protective effect of poor sanitation. This unexpected finding may reflect residual confounding, limitations in sanitation measurement or classification, or differences in the socioeconomic and geographic distribution of households. In particular, if maternal overweight and household DBM are more concentrated in urban and wealthier households, adequacy of toilet facilities may partly capture underlying socioeconomic position rather than an independent relationship with DBM. This pattern may also reflect an emerging socioeconomic gradient in which overweight and obesity become more common among relatively advantaged households while child undernutrition persists [58,59]. This explanation remains tentative and should be examined in future studies.

Maternal age modified the association between women’s empowerment and DBM of mother-child dyads. Specifically, among younger mothers aged 15–19 years, those at either the lowest or highest levels of empowerment were more likely to experience DBM compared to mothers aged 20–34 years old with the highest empowerment level. However, these estimates should be interpreted cautiously because the confidence intervals were wide, suggesting imprecision and possible instability, particularly in the adolescent age group. The observed pattern may also reflect some limitations in measuring empowerment (using the empowerment index) in this age group. Adolescent mothers, given their age-related developmental trajectory, completion of schooling, independent living from their parents, may experience distinct social, economic, and developmental circumstances. Further studies with larger samples of adolescent mothers are needed to clarify this effect-modification.

Consistent with prior work, we found that mother-child dyads in wealthier households had higher odds of DBM compared with those in the poorest households [25]. The positive concentration index indicates that inequality in DBM was concentrated among higher wealth quintiles, with middle, richer, and richest households showing significantly higher odds of DBM than the poorest. One possible explanation is disparities in mothers’ dietary practices: mothers from affluent households may be more inclined to choose meals insufficient in essential micronutrients yet high in fat, and refined sugars, thereby increase their susceptibility to overweight or obesity [25]. These findings suggest that middle- and high-income households in Mozambique may be at a greater risk for DBM, which aligns with findings from previous studies [58,60,61].

Mozambique’s rapid economic expansion before 2015, largely fueled by extractive industries and megaprojects, positioned the country among the fastest-growing economies globally. However, the benefits of this growth were unequally distributed, primarily accruing to the wealthiest 20% of the population, while poverty deepened among the poorest majority. Despite sustained, though slower, growth after 2015, extreme poverty increased, with nearly three-quarters of the population living on less than $2.15 a day by 2019 [62]. This economic paradox of high growth with rising poverty and inequality has important implications for health and nutrition: as incomes and urbanization rise among a minority, dietary transitions toward energy-dense, processed foods are observed, while widespread poverty sustains undernutrition. This coexistence of ‘overnutrition’ in wealthier groups and persistent undernutrition in vulnerable populations underpins DBM in Mozambique, reflecting an ongoing epidemiological and nutritional transition shaped by uneven economic development [40,62–64]. Like many other LMICs, Mozambique has made remarkable progress in curtailing undernutrition rates in the past decade [40,63]. At the same time, rising rates of overweight and obesity, linked to the country’s ongoing epidemiological and nutritional transition, are placing new pressures on the Mozambique National Health System, with infectious diseases and nutrient deficiencies persisting alongside increasing diet-related NCDs. This manifests at both population and household levels [64].

Recent studies underscore a rapid change in the nutritional health status of adults and pregnant women, as well as evolving dietary preferences [65,66]. These studies provide evidence of an ongoing nutrition transition in Mozambique. The shift towards a greater preference for ultra-processed and energy-dense foods and beverages may lead to a diminished intake of essential nutrients and ultimately contribute to child undernourishment. This dietary shift over the past decades underlies the rising prevalence of DBM in Mozambique. Historically, the most prevalent forms of malnutrition among children under five and adolescents worldwide have been undernutrition and micronutrient deficiencies, including stunting, wasting, underweight, and deficiencies of essential micronutrients. Global trends indicate a rapid rise in childhood overweight and obesity, even in countries still grappling with undernutrition conditions. In reality, overweight and obesity are the only forms of malnutrition that are rising among children under five and adolescents at a global level [67,68].

Our findings are valid for Mozambique. However, they can be applied to other LMICs where mother-child dyads experience similar outcomes. The issues we identified will need appropriate interventions to address the detrimental effects of malnutrition in Mozambique. Tackling these issues requires implementing actions that combat all forms of malnutrition, including driving agricultural and food policy research and providing evidence-based policy support to the local government and agencies. One strategy is the adoption of the concept of “double-duty actions,” first introduced in the 2015 Global Nutrition Report (IFPRI, 2015), and gradually used in nutrition research to holistically address this complex public health problem [69]. Double-duty actions encompass interventions, programs, policies, and regulations that simultaneously reduce the risk of undernutrition and overweight, obesity, or diet-related NCDs [5]. Some double-duty actions are not necessarily new initiatives; rather, they include measures that are already in place but need adaptation to address both obesity and undernutrition. These measures should be further enhanced to specifically support households affected by the problem of DBM nationwide.

### Strength and limitations

This study has several limitations. The cross-sectional nature of the data analysed cannot establish causality, only association. The composite definition of DBM may mask heterogeneity among different forms of child undernutrition, as stunting, wasting, and underweight may have different underlying determinants. Separate analyses of these indicators were not conducted because that was not the focus of this study. The findings should be interpreted as reflecting the overall coexistence of maternal overnutrition and child undernutrition. Due to unavailability of the data, various potential confounders (such as physical activity, dietary intake, household food security, cultural influences on caregiving practices and foods, more detailed components of nutritional status like body composition or biochemical or metabolic status) that might affect DBM cannot be included in the analysis. Therefore, further exploration is warranted to ascertain the contribution of these potential determinants to the development of various forms of DBM in Mozambique.

Despite these limitations, the present study has several important strengths. The data were extracted from a nationally representative large, randomized sample, enhances the generalizability of the findings. This study provides national-level evidence on presence of DBM among mother-child dyads in the same households in Mozambique which was not previously reported at the national scale. This study also highlights the wealth-related inequalities in DBM in Mozambique, providing an additional dimension beyond prevalence estimates. Therefore, the findings from this study provides crucial evidence to develop and implement interconnected policies that improve maternal and child health and nutrition in Mozambique.

## Conclusion

Our study identified several factors associated with the nutritional status of women of reproductive age (15–49 years) and children (0–59 months), demonstrating the continued presence of DBM in Mozambique. The coexistence of maternal overweight and child undernutrition within the same household highlights an emerging public health challenge and underscores the complex nature of malnutrition among mother-child dyads. Although the estimated prevalence of DBM was relatively low (5.5%), the findings suggest that multiple forms of malnutrition continue to coexist within the population and are influenced by socioeconomic and demographic factors. The study findings will assist policymakers in identifying individuals at risk of DBM and enabling the development and implementation of targeted programs. Mozambique has a robust policy framework to address malnutrition in all its forms. Strengthening existing maternal and child health and nutrition programs through context-specific double-duty actions, including the integration of interventions that simultaneously address child undernutrition and maternal overweight, may help reduce nutritional inequalities and support progress toward achieving SDG, Target 2.0.

## Supporting information

S1 File S1-S3 Figs, S1-S5 Tables. (DOCX)
